# Diverse Internal Symbiont Community in the Endosymbiotic Foraminifera *Pararotalia calcariformata*: Implications for Symbiont Shuffling Under Thermal Stress

**DOI:** 10.3389/fmicb.2018.02018

**Published:** 2018-09-11

**Authors:** Christiane Schmidt, Raphael Morard, Oscar Romero, Michal Kucera

**Affiliations:** MARUM – Center for Marine Environmental Sciences, University of Bremen, Bremen, Germany

**Keywords:** photosymbiosis, diatoms, benthic foraminifera, symbiont shuffling, thermal stress, invasive species, algae culturing, symbiont flexibility

## Abstract

Many shallow-water tropical and subtropical foraminifera engage in photosymbiosis with eukaryotic microalgae. Some of these foraminifera appear to harbor a diverse consortium of endosymbiotic algae within a single host. Such apparent ability to contain different symbionts could facilitate change in symbiont community composition (symbiont shuffling) and mediate the ecological success of the group in a changing environment. However, the discovery of the intra-individual symbiont diversity was thus far based on symbiont culturing, which provides strong constraints on the vitality of the identified algae but provides poor constraints on their initial abundance and thus functional relevance to the host. Here we analyze the algal symbiont diversity in *Pararotalia calcariformata*, a benthic foraminifera sampled at four stations, inside and outside of a thermal plume in the eastern Mediterranean coast of Israel. This species has recently invaded the Mediterranean, is unusually thermally tolerant and was described previously to host at least one different diatom symbiont than other symbiont-bearing foraminifera. Our results using genotyping and isolation of algae in culture medium, confirm multiple associations with different diatom species within the same individual. Both methods revealed spatially consistent symbiont associations and identified the most common symbiont as a pelagic diatom *Minutocellus polymorphus.* In one case, an alternative dominant symbiont, the diatom *Navicula* sp., was detected by genotyping. This diatom was the third most abundant species identified using standard algae culturing method. This method further revealed a spatially consistent pattern in symbiont diversity of a total of seventeen identified diatom species, across the studied localities. Collectively, these results indicate that *P. calcariformata* hosts a diverse consortium of diatom endosymbionts, where different members can become numerically dominant and thus functionally relevant in a changing environment.

## Introduction

Photosymbiosis is a widespread phenomenon among shallow-dwelling marine organisms and appears to convey strong benefits to the host organisms, facilitating population densities and calcification rates that are unmatched by symbiont-barren relatives (Duguay, 1983; Lee et al., 2010). The evolution of algal endosymbiosis requires multiple adaptations, including immunological recognition and metabolic and reproductive integration. These adaptations lead to opposing selective pressures: metabolic and reproductive integration of the symbiont requires high host specificity, but high specificity comes at the cost of higher vulnerability, because the holobionts then depends on finding its specific symbiont, whose specific environmental adaptations dictate the success of the holobiont (Baker, 2003). As a result, many marine algal symbioses are not strictly specific: for example, corals and some foraminifera are associated with a high genetic diversity of the dinoflagellate *Symbiodinium* (e.g., Schoenberg and Trench, 1980; Momigliano and Uthicke, 2013). In corals, this endosymbiotic associations can comprise eight evolutionarily divergent clades (A–H) of *Symbiodinium* (Coffroth and Santos, 2005). These clades are not only genetically distinct, but also functionally diverse, facilitating ecological adaptations to local environments (Baker and Romanski, 2007; Wilkinson et al., 2015). Symbiont shuffling, the change of symbiont community composition, was shown in corals and provides ecological advantage of coral populations with mixed symbiont communities (Berkelmans and van Oppen, 2006). However, not in all cases endosymbiotic flexibility leads to higher thermal tolerance, so does the environmentally resistant massive coral *Porites* sp. exhibit low symbiont flexibility and harbors a taxonomically narrow *Symbiodinium* assemblages (Putnam et al., 2012).

Only 23% of coral species host multiple zooxanthaellae clades (Goulet, 2006). The maintenance of a diverse internal symbiont pool comes at an energetic cost to the physiology of the coral, which is shown by the fact that corals, which host the thermally tolerant type D zooxanthellae grow less than those which host type C2 zooxanthellae (Jones and Berkelmans, 2010). The coral-algal symbiosis can remain stable over more than 15° of latitude and a range of sea surface temperature profiles (Thomas et al., 2014) or remain constant despite being introduced to new habitats (LaJeunesse et al., 2005). In corals, which host a wider symbiont community composition, the community can change over time or in response to stress event such as bleaching (Little et al., 2004; Cunning et al., 2015; Silverstein et al., 2015). After such an event the relative abundance of the symbionts in the internal pool of an individual can change (Baker et al., 2004; Berkelmans and van Oppen, 2006; Mieog et al., 2007) permanently, or can revert back to the original symbionts post-bleaching (Sampayo et al., 2008). The process that a new symbiont partner is taken up from the environment and positively selected after the changed environmental condition, is described as the adaptive bleaching hypothesis (Buddemeier and Fautin, 1993; Buddemeier et al., 2004). Switching of symbionts is likely limited to early coral larvae or juvenile stages because adult corals have been shown to be unable to form symbiosis with new exogenous algal symbionts (Goulet and Coffroth, 2003; Little et al., 2004; Coffroth et al., 2010). The change of symbiont type after a stress event is likely not limited to corals and the study of this phenomenon in other groups can provide important constraints on the mechanisms by which an internal pool of potentially functional symbionts is maintained. A good model group for such studies are benthic foraminifera, as this group harbors diverse and flexible symbiont associations, involving diatoms, dinoflagellates, chlorophytes, or unicellular rhodophytes (Leutenegger, 1983, 1984; Lee, 1998; Pochon et al., 2001).

Benthic foraminifera are dominant sediment builders in many shallow-water coral-reef environments and thus important contributors to the carbon cycle (Scoffin and Tudhope, 1985; Langer, 2008). The association with diatoms is especially prominent in foraminifera, as four families host them (Lee et al., 1989; Lee, 1992; Lee and Correia, 2005). The diatoms remain “naked” when kept as symbionts, but when cultured, they form frustules, allowing species-level taxonomic identification (Lee et al., 1989; Lee, 1991). They belong to distantly related taxonomic genera, and previous studies spanning over 3,500 isolations in total, showed that six common diatom species were found to be involved in over 75% of all the foraminifera hosts examined: *Nitzschia frustulum var. symbiotica, Nitzschia laevis, Nitzschia panduriformis, Fragillaria shiloi, Amphora roettgerii*, and *A. erezi* (Lee et al., 1995; Lee, 2011). In total at least twenty small diatom (<10 μm) species have been identified as symbionts so far in benthic foraminifera, which are reoccurring in different habitats (Lee et al., 1995). Symbiont-culturing studies revealed that up to three different diatom species can be present inside one host at the same time (Lee, 2011), indicating a considerable flexibility in the association.

The symbiont culturing method (Lee, 1991) allows rapid identification of the potential diatom symbionts of a benthic foraminiferal host. This method has the advantage to only identify only living cells, but the relationship of species abundances in the cultures to the initial concentrations in the foraminifera are not constrained. This means that this method cannot identify which diatom(s) were abundant and thus functionally relevant in the host at the time of its life. It is well-known and accepted by the scientific community that there is the existence of culturing bias in bacteriology (Handelsman, 2004), but little attention has been given to this in benthic protists. Here we combine symbiont culturing with genotyping in a replicated sampling with the aim to determine whether the multiple symbiont-host associations are predictable and whether different members of the symbiont pool can be functionally relevant. We focus our study on the benthic foraminifera *Pararotalia calcariformata*, which is unusually thermally tolerant and where initial results indicated the presence of multiple symbiont types (Schmidt et al., 2015). The species has previously been shown to survive and calcify during summer month in the thermal pollution plume caused by cooling water outlet of the power plant Hadera on the eastern Mediterranean coast of Israel (Arieli et al., 2011; Schmidt et al., 2016b; Titelboim et al., 2016, 2017). Laboratory experiments have confirmed that *P. calcariformata* could survive exposure to 42°C for 3 weeks (Schmidt et al., 2016b). This species thus represents a good opportunity to investigate symbiont diversity within the host and in individuals across the thermal gradient around the Hadera thermal plume.

## Materials and Methods

### Sampling Locations, Collection, and Preparation of Samples

**FIGURE 1:**
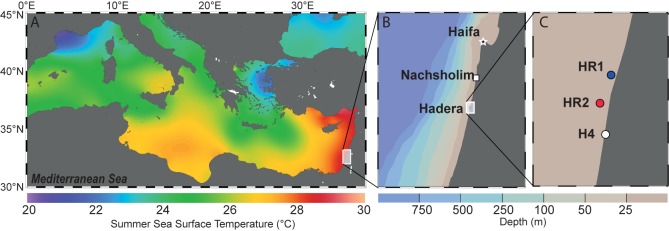
**(A)** Summer Sea Surface Temperatures (SST) in the Mediterranean Sea. The background shows the mean annual SST extracted from the World Ocean Atlas 2013 (Locarnini et al., 2013) and was generated with Ocean Data View (Schlitzer, 2016). **(B)** Israeli coastline showing the two sampling areas. The background shows depth distribution of the sea floor generated with Ocean Data View (Schlitzer, 2016). **(C)** Detailed view of the three locations near the Hadera power plant. HR1, HR2 (R stands for ridge, depth 5–7m) and H4 (depth 0.5 – 2 m).

Samples of living specimens of *P. calcariformata* were collected from four different locations along the Israeli coastline in April and October 2013 (**Figures 1A–C**). Three locations are located in the vicinity of the Hadera power plant, and one location, the Nachsholim National Park, served as a natural control location as it is 18 km north of Hadera, and not influenced by the thermal plume (see **Table 1** for GPS coordinates). Stations with the closed distance from the hot water outlet were HR1 and HR2 (Hadera Ridge 1 and 2). In these two locations, foraminifera were sampled by scuba diving from sediment collected at underwater ridge formations at depth of 5–7 m (**Figure 1C**). Station H4 (Hadera 4) is located further away from the hot water outlet (not shown) and was sampled at the same depth of 0.5–2 m as the control location Nachsholim by snorkeling and beach walking. Temperature regimes in the year 2013 of stations H4 and Nachsholim were recorded in 15 min intervals by data loggers (Onset Hobo, MA, United States) installed at 0.5 m water depth (Titelboim et al., 2016). These were in a similar range as thermal regimes of the natural seawater at the year of collection in Habonim, a monitoring station, near the study locations and were in the range of 15–30°C (Schmidt et al., 2016b). Samples from the field containing living foraminifera attached to sediment and algae were transported in plastic bottles filled with the sample and seawater to the laboratory at Ben Gurion University, Beer Sheva, Israel. There specimens of *P. calcariformata* were individually picked from the bulk sediment material and shipped in plastic jars (volume of 120 mL) filled with seawater and a few grains of substrate sediment by express to the laboratory (MARUM, University of Bremen, Germany), where further extractions and cultures were performed. Cultures of the foraminifera were maintained as previously described (Schmidt et al., 2015) from April 2013 (stations N, HR1, HR2) and from October 2013 (stations N and H4) to November 2013 until specimens were removed from the culture, cleaned from any adhering algae or sediment using a stereomicroscope until further processing. For control station Nachsholim genotyping was conducting for 10 specimens 21 days after collection and on 10 specimen held in culture for 6 months. The results of genotyping from freshly collected foraminifera and foraminifera held in culture were similar (**Table 1**). Hence, we assume that culturing minimally changed their symbiont community.

**Table 1:** Sampling stations of the study and foraminifera or diatom cultures analyzed with the respective techniques: genotyping and standard algae culturing.

Station name	Latitude (°N)	Longitude (°E)	Depth (m)	Sampling Date	*N* genotyping	*N* symbiont culturing
N	32.623611	34.919833	0.5–2	15.04.13	12	
N	32.623611	34.919833	0.5–2	23.10.13	9	10
H4	32.446967	34.878000	0.5–2	20.10.13	10	10
HR1	32.46110	34.87955	5–7	15.04.13	9	10
HR2	32.45442	34.87652	5–7	15.04.13	9	10

### DNA Genotyping: Extraction, Cloning, and Sequence Identification

**FIGURE 2:**
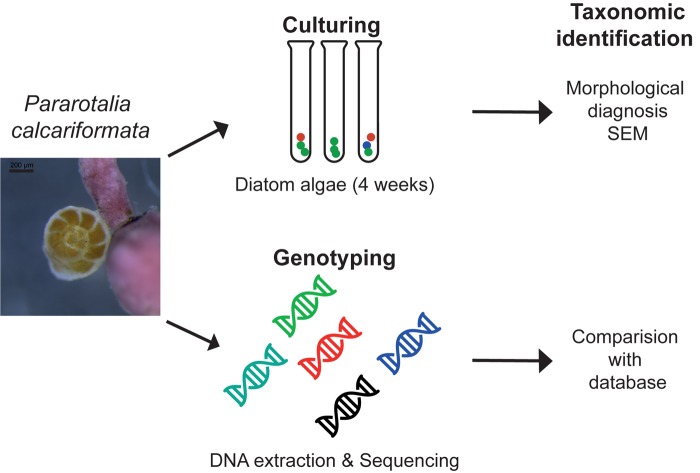
Methodological approach isolating the diatom symbiont community of benthic foraminifera with the standard culturing approach and genotyping as conducted in this study.

Fifty specimens of cultivated *P. calcariformata* were individually isolated into 50 μl of GITC^∗^ extraction buffer and DNA was extracted (**Figure 2**) as in Schmidt et al. (2015). Amplification of a fragment of 400 bp of the 3′ end of the SSU rDNA of the symbionts of *P. calcariformata* was carried out with the GoTaq polymerase (Promega) using the forward primer SymSF1 (5′-GGTTAATTCCGTTAACGAACGAGA-3′) that anneal with all eukaryotes except foraminifera coupled with the universal reversed primer 1528R (5′-TGATCCTTCTGCAGGTTCACCTAC-3′) (Medlin et al., 1988). The PCR mix was composed of 3 μl of 5× green buffer (final concentration 1×), 0.15 μl of each primer at 10 μM (final concentration: 0.1 μmol/μl), 1.5 μl of MgCl2 at 25 μM (final concentration: 2.5 μmol/μl), 0.6 μl of dNTP mix at 10 μM (final concentration: 0.4 μmol/μl), 0.15 μl of GoTaq at 5 U/μl (final concentration: 0.05 U/μl), and 8.45 of MilliQ water for a final volume of 14 μl. The PCR mix was prepared under a UV hood and the 1 μl of sample DNA was loaded under a different hood using filter tips. PCR amplification conditions were as follows: initial denaturation at 95°C for 2 min followed by 35 cycles at 95°C for 30 s, 56°C for 30 s and 72°C for 30 s, and 2 min of final extension at 72°C. After the end of the cycling, 4 μl of each PCR product was migrated on 1.5% agarose gel to check if the PCR was positive and a single band was observed in 47 positive PCR products (3 products were negative or too weak for sequencing). For the positive PCR products, the 11 μl remaining were purified on column using the QIAquick PCR purification kit (QIAGEN) and directly sequenced by an external provider (LGC Genomics, Berlin). The 47 obtained chromatograms were carefully checked and no sign of multiple signals was detected in 39 of them. Among the eight chromatograms displaying multiple signals, one specimen from each location (4) was selected for cloning. The cloning was performed using Zero Blunt^®^ TOPO^®^ PCR Cloning Kit (Invitrogen) with TOP10 chemically competent cells following manufacturer’s instructions. The obtained clones were purified using the PureLink^TM^ HQ Mini Plasmid Purification Kit (Invitrogen) and sequenced. Between 22 and 24 sequences were obtained per individual foraminifera. The resulting sequences were compared to the SILVA database using the SINA 1.2.11 alignment tool (Pruesse et al., 2012), with the “Search and Classify” option with a rejection threshold of 60% has and a number of 10 neighbors. The SINA returned partially resolved ranked taxonomy of each sequences based on the taxonomy of the nearest neighbors found in the SILVA database (**Figure 2** and **Supplementary Table 1**).

### Symbiont Culturing (SC): Isolation of Diatoms and Identification by Scanning Electron Microscopy

Forty specimens of *P. calcariformata* (10 from each location, **Table 1**) were individually cleaned from epiphytic algae with sterilized brushes and crushed open in glass petri dishes. The protocol by Lee (1991) for isolating diatoms in axenic media from foraminifera was followed. Cultures were grown for 4 weeks (**Figure 2**) (07/11/2013–06/12/2013) and illuminated at 30– 50 μ mol photons m^2^s^−1^ (1,000 K:420 nm, 12 h dark: 12 h light). Cultures were concentrated by removing 4 mL of culture media and vortexed. Aliquots of 4 mL of the original culture and the bottom culture (culture vial with 2 mL culture medium included a small biofilm of diatoms on bottom, which is referred to as bottom culture) were incubated at 60°C overnight with H_2_O_2_. H_2_O_2_ was added at a concentration of 1:1 to the cultures to oxidize organic material and to clean the frustules for SEM preparations. Each culture/H_2_O_2_ mix was filtered over a membrane filter (Whatman, Nucleopore Track Etch Membrane Filters, 2 μm), which was mounted on SEM stubs (scanning electron microcopy) and accordingly labeled. For each of the 40 cultures, two SEM stubs were prepared (4 mL aliquot/2 mL bottom culture). Up to 15 SEM pictures of representative specimens were taken per stub, specimens were identified to species level following the taxonomy of Round et al. (1990) and previous work on diatoms in benthic foraminifera (Lee et al., 1989, 1995; Lee, 1991; Lee and Correia, 2005). In cases were newer taxonomic names were given since the work of Lee et al. (1989, 1995); Lee (1991), we updated the taxonomy of endosymbiotic diatoms in foraminifera, but refer here to both names. These are as follows (new vs. old name): *Halamphora kolbeii* (Aleem) = *Amphora bigibba* (Álvarez-Blanco and Blanco, 2014), *Halamphora subsalina = Amphora tennermia* (Levkov, 2009), *Tryblionella coarctata f. densestriata = Nitzschia panduriformis* (Álvarez-Blanco and Blanco, 2014), *Astartiella punctifera* (Hustedt) = *Achnanthes punctifera (Hustedt)* (Moser et al., 1998). Photographic material and taxonomic identification of all specimens are deposited under doi: 10.3389/fmicb.2018.02018 on Figshare. Representative specimens of each identified species are shown in **Figure 4**.

## Results

### DNA Genotyping

**FIGURE 3:**
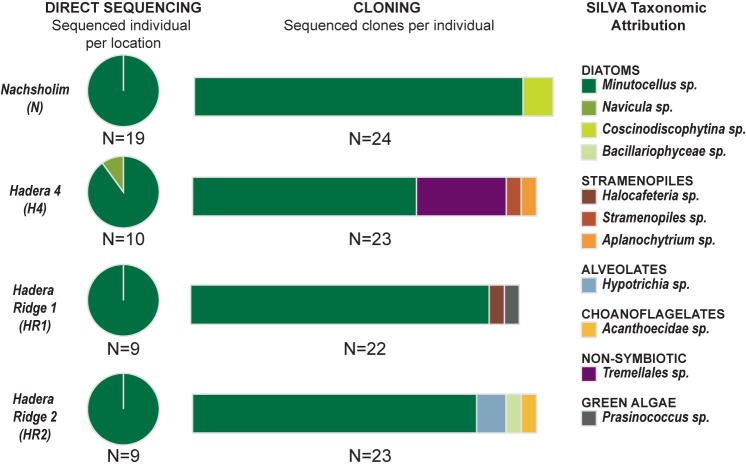
Genetic Results. **(Left)** Indicates the results obtained with direct sequencing for each of the four locations. **(Central)** Indicates the results of cloning obtained for one selected individual for each location. **(Right)** Indicates the taxonomic assignation of the sequences using the SILVA database.

Out of the 50 single-specimen DNA extractions, 47 yielded strong positive PCR products (**Table 1**). These were directly sequenced, as no multiple bands were visible on the gel after migration. All sequences yielded a clean dominant signal on the obtained chromatograms, except for eight sequences, where weaker sub-signals were observed. Comparison with the SILVA database revealed that 46 sequences, except one, were attributed to the diatom species *Minutocellus polymorphus* with the level of identity of 92–95% (**Figure 3** and **Supplementary Table 1**). The remaining sequence belongs to the diatom *Navicula* with 98% identity. Of the specimens which yielded multiple signals on the chromatograms, potentially due to the presence of multiple symbiont strains, four (one for each location) were used to build clone libraries to obtain a better representation of the symbiont community composition, approximating relative proportions within single individuals. We obtained between 22 and 24 clone sequences per specimen (92 clone sequences in total). For each clone library, 65–91 % of the sequences belong to *M. polymorphus* (**Figure 3**). Multiple other potential symbiont strains belonging to diatoms (*Coscinodiscophytina* sp., *Bacillariophyceae* sp.), green algae *Prasinococcus*, other stramenopiles than diatoms (*Halocafeteria* sp., *Aplanochytium* sp., *Straemenopiles* sp.) and alveolates (*Hypotrichia* sp.) were identified. We also identified sequences of non-symbiotic marine organisms such as choanoflagellates, as well as one individual sequence belonging to a jelly fungus (order Tremellales) from the terrestrial environment (likely indoor airborne contaminant).

### Symbiont Culturing

**FIGURE 4:**
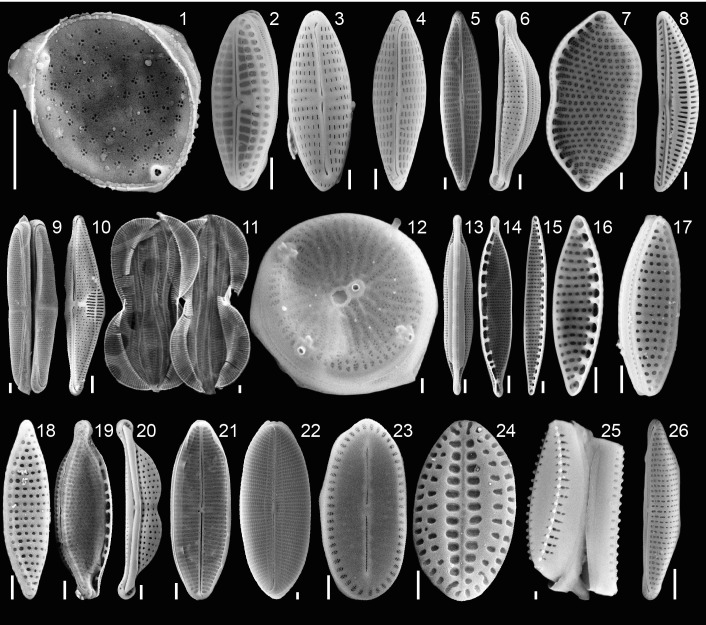
Plate of isolated endosymbiotic diatoms from the benthic foraminifera *P. calcariformata*, specimens ordered by total abundance over all isolations in this study. Scale bar 1 μm, 1, *Minutocellus polymorphus*; 2, *Olifantiella pseudobiremis*; 3, *Navicula* sp., 4, *Navicula cf. salinicola*; 5, *Navicula duerrenbergiana*; 6, *Halamphora subsalina*; 7, *Tryblionella coarctata f. densestriata*; 8, *Amphora inariensis*; 9, *Amphora jostesorum*; 10, *Amphora pseudoeximia*; 11, *Entomoneis paludosa*; 12, *Minidiscus* sp., 13, *Nitzschia* sp.; 14–18, *Nitzshia frustulum var. symbiotica*; 19, *Nitzschia microcephala*; 20, *Halamphora kolbeii*; 21, *Astartiella punctifera*; 22, *Diploneis* sp.; 23, *Cocconeis* sp.; 24, *Amphicocconeis* sp.; 25, *Pseudostaurosira* sp.; 26, *Cymbella* sp. For taxonomic references please see section “Materials and Methods.”

All 40 symbiont extraction cultures grew and yielded identifiable diatoms (**Tables 1**, **2** and **Figure 4**). The most abundant species, was *Minutocellus polymporphus*, found in 73% of the isolations (**Table 2**). The next common symbionts were *Navicula* spp. and *Olifantienlla pseudobiremis*, which were identified in about half of the isolated specimens (43–50%) (**Table 2**). *Nitzschia panduriformis*, *Amphora tenerrmia*, and *Nitzschia frustulum* var. *symbiotica* Lee and Reimer emend. were identified in 25–35% of the isolated specimens. Another seven small diatom species were identified in <18% of the specimens (**Table 2**). Two larger diatom species in the size range of 18–25 μm (*Nitzschia* sp. and *Entomoneis* sp.) were identified in 8% of the specimens. **Supplementary Table 2** lists all symbionts identified per culture.

**Table 2:** Endosymbiotic diatoms isolated from the benthic foraminifera *Pararotalia calcariformata*.

	Station	N	H4	HR1	HR2
	
	Depth	0.5–2 m		5–7 m	
	Sampling date	20.10.13		15.04.13	

**Species name**	**% occurance of total**	**% occurance per location**			
*Minutocellus polymorphus*	73	90	90	70	40
*Olifantiella pseudobiremis*	50	0	20	80	100
*Navicula* spp.	43	30	20	20	100
*Halamphora subsalina*	35	10	40	30	50
*Tryblionella coarctata* f. *densestriata*	35	20	10	30	80
*Nitzschia frustulum* var. *symbiotica*	28	0	20	30	60
*Amphora* spp.	20	20	0	0	60
*Entomoneis paludosa*	18	0	0	60	10
*Minidiscus* sp.	18	0	0	0	70
*Nitzschia* spp.	15	0	10	10	40
*Halamphora kolbeii*	10	10	0	10	20
*Astartiella punctifera*	10	40	0	0	0
*Diploneis* sp.	8	0	0	0	30
*Cocconeis* sp.	3	0	0	0	10
*Amphicocconeis* sp.	3	0	0	0	10
*Pseudostaurosira* sp.	3	0	0	0	10
*Cymbella* sp.	3	0	0	0	10

*Species names listed in order of abundance from all isolated specimens. Species names have been summarized by genus in these cases: Navicula spp. (Navicula cf. salinicola and Navicula duerrenbergiana), Amphora spp. (Amphora inariensis, Amphora jostesorum, Amphora sp., and Amphora pseudoeximia) and Nitzschia spp. (Nitzschia sp. and Nitzschia microcephala), Taxonomy is based on these publications (Lee et al., 1989, 1995; Round et al., 1990; Lee, 1991; Lee and Correia, 2005), in some cases names were updated (new name = old name): Halamphora kolbeii (Aleem) = Amphora bigibba (Álvarez-Blanco and Blanco, 2014), Halamphora subsalina = Amphora tennermia (Levkov, 2009), Tryblionella coarctata f. densestriata = Nitzschia panduriformis (Álvarez-Blanco and Blanco, 2014), Astartiella punctifera (Hustedt) = Achnanthes punctifera (Hustedt) (Moser et al., 1998).*

**FIGURE 5:**
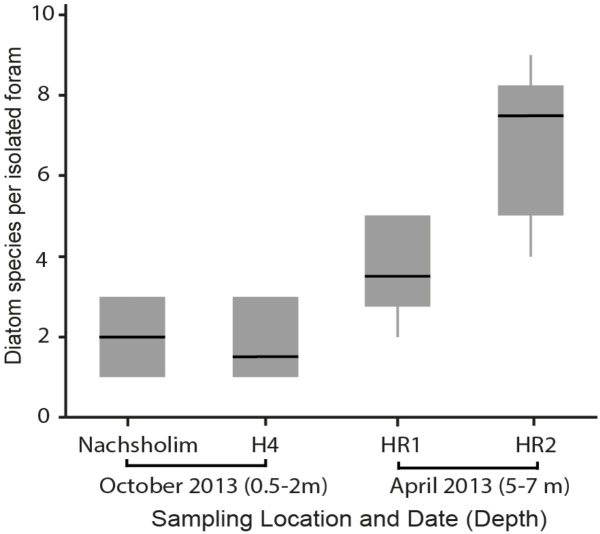
Diatom diversity found in isolations of the benthic foraminifera *P. calcariformata* performed for this study (*N* = 40), specimens were obtained from the locations affected by the heat plume (H4, HR1, HR2) and the control station (Nachsholim) at respective sampling dates and depth.

Among the four sampled locations, the deeper stations (HR1, HR2), sampled in spring, showed a higher diatom diversity than the shallow stations (Nachsholim, H4) sampled in autumn (**Table 1**). Foraminifera from HR1 and HR2 yielded on average four to eight different diatom species at the same time, whereas individuals from the shallower stations yielded on average two different diatom species (**Figure 5**). *Minutocellus polymorphus* was the dominant symbiont type at all stations, except in one station HR2, where *Navicula* sp. was the dominant symbiont, followed closely by *M. polymorphus*.

## Discussion

Our results confirm the presence of infections of multiple different diatoms in the benthic foraminifera *P. calcariformata.* Here we identified seventeen different symbionts associated with *P. calcariformata*. This is within the range of the diversity of diatoms that are known as foraminifera symbionts isolated from the many locations worldwide including the Mediterranean to date (Lee, 2011). In previous symbiont culturing studies, *Nitzschia frustulum* var. *symbiotica* Lee and Reimer emend. 2001 was the most commonly isolated diatom, occurring in about 1/3 of the isolation (Lee et al., 1995; Lee, 2006). In our material, this species was identified in only 25% of the isolations, which could also have to do that the study by Schmidt et al. (2015) was the first to describe it as endosymbionts in the species *P. calcariformata*. The most commonly isolated symbiont in this data set was again the pelagic diatom *Minutocellus polymorphus* (Hargraves and Guillard) Hasle, Stosch, and Syvertsen and the second most common isolate was the diatom *Navicula* sp. whereas *Navicula* sp. has been identified as symbiont in foraminifera before (Lee et al., 1980). The dominant occurrence of *M. polymorphus* confirms the initial results by Schmidt et al. (2015) and supports the identification of this diatom as a previously unrecognized foraminiferal symbiont. Despite its small size (∼3 μm diameter), we do not consider it likely that this species has been overlooked as the isolation protocol by Lee (1991) uses filters with pores of 3 μm diameter as well. *Minutocellus polymorphus* is found free-living in the Mediterranean (Sarno et al., 1993). The species has been identified as cosmopolitan (Hasle, 1983; Malviya et al., 2016) and is found to form phytoplankton blooms in China (Qiao et al., 2017). Due to its small size and difficulty of detection, this nanophytoplankton (2–20 μm) is poorly characterized but has been lately described in the northwestern Mediterranean Sea to occur massively in spring blooms reaching the sea floor in high sinking rates (Leblanc et al., 2018). This study reported the diatom genus *Minidiscus* to form the spring blooms, and a genus which was also isolated in this study from *P. calcariformata* (**Table 2**).

*Pararotalia calcariformata* next to the diatom *M. polymorphus* on average up to two other diatom species in the control stations Nachsholim and H4, indicating intra-individual symbiont diversity. We detected this by genotyping less variable symbionts and by isolating symbiont cultures more, which could not be a culture artifact, because samples have been treated the same. Within the four specimens which have been extensively cloned, only two yielded two different diatom sequences (*Minutocellus* and *Coscinodiscophtina* for the specimen cloned in Nascholim, *Minutocellus* and *Bacillariophycae* sp. for the specimen from Hadera Ridge). This compares to gentoytpying of symbionts of other larger benthic foraminifera containing diatoms, such as *Amphistegina lessonii*, which yielded in six haplotypes of two lineages of diatoms of the order Fragilares (Stuhr et al., 2018b). *A. lobifera* from the Red Sea hosted 70% diatoms belonging to the order Fragilares and did not seem to exhibit preference for one symbiont type (Schmidt et al., 2016a). In the contrary, *Amphisegina gibbosa* from the Florida keys host a narrower symbiotic composition of a single diatom sequence type (Stuhr et al., 2018a). A study from the Great Barrier Reef found also consistent photo-symbiotic taxa of *A. lobifera*, which were also mainly belonging to the order Fragilariales (Naviculales and Bacillariales being in the 5–10% range), which was consistent among a gradient of different reef sites but varying communities of procaryotes in their microbiome (Prazeres et al., 2017).

In comparision to *Amphisteinga* spp. the symbiont diversity in *P. calcariformata* seems to be higher. The culturing results in the deeper locations was higher and in one location (HR2) showed up to nine different diatom species have been detected by symbiont culturing inside a single host. It should be noted that HR1 and HR2 are also the stations closes to the heat-plume water outlet, but they were also collected slightly deeper than other samples, which could also have an effect on their symbiont composition. This is the highest intra-individual symbiont diversity from a benthic foraminifera reported to date, and was not observed by the foraminifera isolated by Lee et al. (2011), who found a maximum of three diatoms per host. These results imply that the diatom symbiosis in *P. calcariformata* is flexible and different members of the internal pool can be numerically dominant (as revealed by direct sequencing).

The existence of a diverse internal symbiont pool in foraminifera implied by our results is significant for their potential role in symbiont shuffling. This is important, because previous experiments failed to provide supporting evidence for symbiont switching as a mechanism of adaptation to environmental change. So far, *in situ* experiments in the Gulf of Eilat showed that foraminifera can be re-infected by symbionts after bleaching, but the reinfection was limited to diatom strains which have been previously isolated from foraminifera hosts (Lee et al., 1986). Planktonic foraminifera have also been shown to be able to take up symbionts after bleaching with the herbicide DCMU, but the symbionts were also taken from algal cultures extracted from the same host population (Bé et al., 1982). Thus, there is no evidence that adult foraminifera are able to obtain “environmental” diatoms as symbionts. Symbionts are characterized by a specific protein “recognition” signature (Chai and Lee, 1999, 2000), which prevents digestion by the host. It is possible that successful infection of foraminifera requires symbionts which express this protein, making environmental infection unlikely, at least in adult stages, as is observed in corals (Goulet and Coffroth, 2003; Little et al., 2004).

Instead, the presence of a diverse internal symbiont pool may provide an effective mechanism for adaptation to stress, allowing “shuffling” by regulating the reproductive success of the pre-existent symbiont species. Studies on two different diatoms in power-plant related heat plumes revealed that *Amphora coffeaeformis* is particularly thermally tolerant (Rajadurai et al., 2005). The genus *Amphora* was also detected in larger benthic foraminifera, in this study (**Figure 3**) and was described as a secondary diatom occurring in larger benthic foraminifera from the Red Sea (Lee et al., 1980). An ability to host a diverse symbiont consortium, including such heat-tolerant diatoms, would be an advantage in a situation where during the life of an individual, environmental conditions reach extreme values. This situation is known to apply to *P. calcariformata*, which seems to reproduce in spring and whose adults have to persist during the summer heat amplified by the excess warmth from the Hadera power plant heat plume (Titelboim et al., 2016, 2017). Our results by genotyping and symbiont culturing confirm that multiple diatom symbionts are functionally relevant in *P. calcariformata*, as they are present during its life, which may allow this species to adapt its symbionts in response to stress. Further testing needs to reveal whether the change of relative proportions of symbionts over time in response to a stress event can cause the unique thermal tolerance of this species observed to date (Schmidt et al., 2016b; Titelboim et al., 2017). Our data in this paper showed that there is so far no difference between the plume environment and the control location, as dominating symbiont *M. polymorphus* was consistent and numerically most abundant throughout.

## Data Availability

The raw datasets of the genetic sequences and the symbiont culturing are attached in the **Supplementary Material**. Sanger sequences were deposited on Genbank under accession numbers MH721032 – MH721170. Image files of diatoms (.zip) and identifier table of diatom images with subsequent taxonomic names are availble on FigShare (https://figshare.com/articles/Original_Data_to_MS_Front_Microbiol_doi_10_3389_fmicb_2018_02018/6965261).

## Author Contributions

CS performed sampling, laboratory work of the isolations, took microscopy images of diatoms, and analyzed the data. CS and OR identified diatom images. RM performed laboratory work on genetic fingerprinting and data analysis. CS, RM, and MK wrote the manuscript. All authors reviewed and approved the final manuscript.

## Conflict of Interest Statement

The authors declare that the research was conducted in the absence of any commercial or financial relationships that could be construed as a potential conflict of interest. The reviewer NK and handling Editor declared their shared affiliation.
